# Successful treatment of transfusion-dependent β-thalassemia: multiple paths to reach potential cure

**DOI:** 10.1038/s41392-025-02135-9

**Published:** 2025-02-17

**Authors:** Michael Morgan, Axel Schambach

**Affiliations:** 1 Institute of Experimental Hematology, Hannover Medical School, Hannover, 30625 Germany; 2 REBIRTH Research Center for Translational Regenerative Medicine, Hannover Medical School, Hannover, 30625 Germany; 3 Division of Hematology / Oncology, Boston Children’s Hospital, Harvard Medical School, Boston, MA 02115 USA

**Keywords:** Molecular medicine, Genetics research

In a recent article in *The Lancet*, an international team led by JL Kwiatkowski, F Locatelli and AA Thompson presented their successful multicenter phase 3 clinical trial (HGB-212, NCT03207009) that used gene therapy to treat severe genetic forms of transfusion-dependent β-thalassemia.^1^ This ground-breaking study showed that the gene therapy cell product (betibeglogene autotemcel or beti-cel) led to transfusion independence in most treated patients (16/18, 89%), all of whom had genotypes that are associated with severe β-thalassemia (i.e., β^0^/β^0^, β^0^/β^IVS1-110^, β^IVS1-110^/β^IVS1-110^).

The different types of thalassemia are named for the type of globin chain of hemoglobin that is defective, such as α-, β-, δβ- and γδβ-thalassemia. Patients suffering from β-thalassemia are most likely to require regular blood transfusions and these transfusion-dependent thalassemia (TDT) patients also need iron chelation therapy to prevent complications like organ damage due to iron overload. Mutations in the hemoglobin beta (HBB) gene that result in reduced or complete ablation of β-globin production lead to insufficient levels of functional hemoglobin. This causes hemolysis and reduced production of erythrocytes due to the imbalance of α- and β-globin proteins. Thus, restoration of the balance of α- and β-globins is a strategy to treat β-thalassemia.

**Fig. 1:**
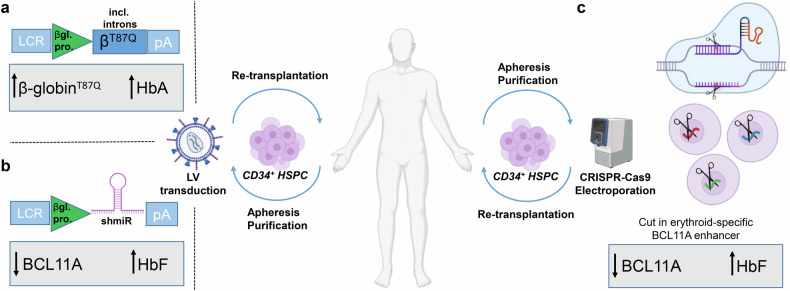
Autologous settings for application of targeted therapies to treat β-thalassemia or sickle cell disease due to β-globin gene mutations. The current work used a SIN lentiviral vector for ex vivo delivery of the T87Q variant of the β-globin gene to the hematopoietic stem and progenitor cells (HSPC) to treat patients with genotypes associated with severe forms of β-thalassemia (**a**). As an alternative strategy to treat patients with β-globin gene mutations, increased fetal hemoglobin (α_2_γ_2_, HbF) was achieved by knock-down of BCL11A using a SIN lentiviral vector for ex vivo delivery of a short hairpin microRNA (shmiR) to HSPC harvested from patients with severe sickle cell disease (**b**). Similarly, ex vivo application of CRISPR-Cas9 genome editing to knock-out BCL11A in HSPC was shown to be another feasible strategy to elevate fetal hemoglobin levels as a treatment strategy for β-thalassemia patients (**c**). Figure created with BioRender.com. LCR locus control region, βgl pro. β-globin promoter, pA polyA signal, HbF fetal hemoglobin, HbA adult hemoglobin

Beti-cel is generated by ex vivo transduction of autologous CD34^+^ hematopoietic stem and progenitor cells HSPC) with the lentiviral vector BB305, which is a replication defective, self-inactivating (SIN), third-generation HIV-1 based lentiviral vector engineered to deliver a variant of the human β-globin gene modified at codon 87 (T87Q) (Fig. 1). This β-globin gene variant was found to be mostly responsible for inhibiting HbS polymerization found in sickle cell disease, the α_2_β^T87Q^_2_ tetramer has similar oxygen affinity as HbA and the β^T87Q^ variant can be used to track gene correction efficiency in clinical trials.^2^

The authors used a hyper-transfusion regimen for at least 60 days prior to HSPC mobilization to maintain hemoglobin levels ≥11 g/dL as they also did in earlier clinical studies for β-thalassemia (HGB-204, HGB-205).^3^ In the current work, an optimized manufacturing process with improved transduction efficiencies was achieved, with mean vector copy number (mVCN) in peripheral blood 24 months after treatment of 1.992 (0.15–3.49, *n* = 16) in patients who became transfusion-independent, as compared to mVCN of 0.7 (0.3–1.5, *n* = 18) in HGB-204 and 1.3 (0.8–2.1, *n* = 4) in HGB-205. The higher transduction efficiencies resulted in total unsupported hemoglobin levels of 10.63 g/dL (9.6–14.0, *n* = 16) in the transfusion-independent patients, the majority of which corresponded to the therapeutic transgene HbA^T87Q^ (9.083: 4.68–12.43 g/dL, *n* = 16). While the earlier clinical trials also had good response rates in consideration of patients who achieved transfusion independence, the patient cohorts treated in those studies had predominantly less severe β-thalassemia genotypes with 44% (*n* = 8 of 18 patients) and 25% (*n* = 1 of 4 patients)^3^ as compared to the current study in which all 18 patients treated had severe genotypes.^1^ The authors suggest that the higher mVCN contributed to a better therapeutic effect with regard to hemoglobin expression. The two patients who did not achieve transfusion independence in this study, one male and one female, had genotypes β^0^/β^0^ and β^IVS1-110^/β^IVS1-110^, respectively. In contrast to the higher mVCN detected in the patients who achieved transfusion independence, these two patients had mVCN of 0.139 and 0.328, with no or 2.52 g/dL total unsupported hemoglobin, respectively. Since the mVCN of the cell product that was infused into these two patients was 1.9 and 2.1, the relatively low mVCN determined 24 months later in the peripheral blood of these two patients indicate that the modified cells may have failed to properly engraft, survive and/or produce β-globin (and in consequence hemoglobin), and were therefore unable to meaningfully contribute to hematopoiesis and support correction of the disease.

Alternative strategies to treat β-thalassemia patients have also shown promise, including use of genome editing technologies such as clustered regularly interspaced short palindromic repeats (CRISPR)-Cas9 (Fig. 1). For example, CRISPR-Cas9 editing to disrupt the BCL11A Erythroid Enhancer in HSPC was shown to reactivate production of fetal hemoglobin (HbF: α_2_γ_2_) in place of adult hemoglobin (HbA: α_2_β_2_), and to overcome the transfusion-dependence in 91% (32/35) of β-thalassemia patients in an international phase 3 trial comprising 20 (57%) TDT patients with severe genotypes^4^ and in 2/2 pediatric β^0^/β^0^ TDT patients in another recent clinical trial^5^. In 2023, the European Medicines Agency (EMA) approved this gene editing therapy^6^ to treat β-thalassemia and severe sickle cell disease. Another study showed that knock-down of BCL11A mRNA expression using an RNA interference strategy that was delivered into autologous CD34^+^ HSPC via a third-generation SIN lentiviral vector was safe and feasible to treat sickle cell disease, which is also caused by mutation of the β-globin gene (Fig. 1).^7^ Expression of the therapeutic transgene, a microRNA-adapted short hairpin RNA, was controlled by β-globin locus regulatory elements for improved expression in the erythroid lineage. The therapy resulted in stable vector integration (copy numbers of 0.23–1.99 in peripheral erythroid cells), transfusion independence in five of six patients and absence of vaso-occlusive events in all patients. While the BCL11A knockdown approach remains to be tested in β-thalassemia, this may represent a useful approach for disease control.

As an outlook towards further advancements for treatment of patients suffering from β-thalassemia and/or other diseases caused by defective β-globin, the lentiviral vector used in the current study could be combined with cell-targeted approaches for delivery of the therapeutic transgene to HSC in vivo. Such approaches are expected to make gene therapies more available due to lower overall costs and elimination of advanced cell processing units currently needed to generate the ex vivo modification and expansion of gene-modified cell therapies. Eventually, such advances will help accelerate delivery of gene therapies across socio-economic and geographic boundaries for improved distribution to the patients most in need.

In conclusion, this pivotal work described by Kwiatkowski and colleagues represents the first phase 3 clinical study using lentiviral gene therapy to introduce functional copies of a modified form of the β-globin gene (β A-T87Q-globin gene) into HSC isolated from patients with severe genetic forms of β-thalassemia. The ongoing follow-up study (LTF-303, NCT02633943) will provide insight into long-term safety and efficacy data for these patients for 15 years after application of the cell product beti-cel, which will help to further direct development of the gene therapy field.

## References

[1] Kwiatkowski, J. L. et al. Betibeglogene autotemcel gene therapy in patients with transfusion-dependent, severe genotype β-thalassaemia (HGB-212): a non-randomised, multicentre, single-arm, open-label, single-dose, phase 3 trial. *Lancet* **404**, 2175–2186 (2024). 39527960 10.1016/S0140-6736(24)01884-1

[2] Pawliuk, R. et al. Correction of sickle cell disease in transgenic mouse models by gene therapy. *Science* **294**, 2368–2371 (2001). 11743206 10.1126/science.1065806

[3] Thompson, A. A. et al. Gene Therapy in Patients with Transfusion-Dependent β-Thalassemia. *N. Engl. J. Med.* **378**, 1479–1493 (2018). 29669226 10.1056/NEJMoa1705342

[4] Locatelli, F. et al. Exagamglogene Autotemcel for Transfusion-Dependent β-Thalassemia. *N. Engl. J. Med.* **390**, 1663–1676 (2024). 38657265 10.1056/NEJMoa2309673

[5] Fu, B. et al. CRISPR-Cas9-mediated gene editing of the BCL11A enhancer for pediatric β0/β0 transfusion-dependent β-thalassemia. *Nat Med*. **28**, 1573–1580 (2022). 35922667 10.1038/s41591-022-01906-z

[6] European Medicines Agency. First gene editing therapy to treat beta thalassemia and severe sickle cell disease. https://www.ema.europa.eu/en/news/first-gene-editing-therapy-treat-beta-thalassemia-and-severe-sickle-cell-disease (2023).

[7] Esrick, E. B. et al. Post-Transcriptional Genetic Silencing of BCL11A to Treat Sickle Cell Disease. *N. Engl. J. Med.* **384**, 205–215 (2021). 33283990 10.1056/NEJMoa2029392 PMC7962145

